# Analysis of P(v-a)CO_2_/C(a-v)O_2_ Ratio and Other Perfusion Markers in a Population of 98 Pediatric Patients Undergoing Cardiac Surgery

**DOI:** 10.3390/jcm12175700

**Published:** 2023-09-01

**Authors:** Matteo Taiana, Irene Tomasella, Alessandro Russo, Annalisa Lerose, Marcello Ceola Graziadei, Luisa Corubolo, Jacopo Rama, Vittorio Schweiger, Alessandro Vignola, Enrico Polati, Giovanni Battista Luciani, Francesco Onorati, Katia Donadello, Leonardo Gottin

**Affiliations:** 1 Cardiothoracic and Vascular Intensive Care Unit, Hospital and University Trust of Verona, P. le A. Stefani, 37124 Verona, Italy; irene.tomasella@aovr.veneto.it (I.T.); alessandro.russo@aovr.veneto.it (A.R.); marcello.ceolagraziadei@aovr.veneto.it (M.C.G.); luisa.corubolo@aovr.veneto.it (L.C.); jacoporama93@gmail.com (J.R.); leonardo.gottin@univr.it (L.G.); 2 Anesthesia and Intensive Care Unit, Magalini Hospital ULSS 9 Scaligera, Villafranca, 37069 Verona, Italy; annalerose@hotmail.com; 3 Anesthesia and Intensive Care Unit, Policlinico G.B. Rossi, Hospital and University Trust of Verona, P. le L. Scuro, 37129 Verona, Italy; vittorio.schweiger@aovr.veneto.it (V.S.); enrico.polati@aovr.veneto.it (E.P.); katia.donadello@aovr.veneto.it (K.D.); 4 Emergency Medicine Department, Hospital and University Trust of Verona, P. le A. Stefani, 37126 Verona, Italy; 5 Cardiac Surgery Unit, Hospital and University Trust of Verona, P. le A. Stefani, 37126 Verona, Italy; giovanni.luciani@aovr.veneto.it (G.B.L.); francesco.onorati@aovr.veneto.it (F.O.)

**Keywords:** venoarterial carbon dioxide difference, congenital heart disease, cardiopulmonary bypass, postoperative care, physiologic monitoring, infant

## Abstract

Background: The so-called Low Cardiac Output Syndrome (LCOS) is one of the most common complications in pediatric patients with congenital heart disease undergoing corrective surgery. LCOS requires high concentrations of inotropes to support cardiac contractility and improve cardiac output, allowing for better systemic perfusion. To date, serum lactate concentrations and central venous oxygen saturation (ScVO_2_) are the most commonly used perfusion markers, but they are not completely reliable in identifying a state of global tissue hypoxia. The study aims to evaluate whether the venoarterial carbon dioxide difference/arterial-venous oxygen difference ratio [P(v-a)CO_2_/C(a-v)O_2_] can be a good index to predict the development of LCOS in the aforementioned patients, so as to treat it promptly. Methods: This study followed a population of 98 children undergoing corrective cardiac surgery from June 2018 to October 2020 at the Department of Cardiac Surgery of University Hospital Integrated Trust and their subsequent admission at the Postoperative Cardiothoracic Surgery Intensive Care Unit. During the study, central arterial and venous blood gas analyses were carried out before and after cardiopulmonary bypass (CPB) (pre-CPB and post-CPB), at admission to the intensive care unit, before and after extubation, and at any time of instability or modification of the patient’s clinical and therapeutic conditions. Results: The data analysis shows that 46.9% of the children developed LCOS (in line with the current literature) but that there is no statistically significant association between the P(v-a)CO_2_/C(a-v)O_2_ ratio and LCOS onset. Despite the limits of statistical significance, however, a 31% increase in the ratio emerged from the pre-CPB phase to the post-CPB phase when LCOS is present. Conclusions: This study confirms a statistically significant association between the most used markers in adult patients (serum lactate concentration, ScVO_2_, and oxygen extraction ratio—ERO_2_) measured in the pre-CPB phase and the incidence of LCOS onset, especially in patients with hemodynamic instability before surgery.

## 1. Introduction

Low Cardiac Output Syndrome (LCOS) is a clinical condition caused by a transient decrease in systemic perfusion secondary to a myocardial dysfunction. The result is an oxygen delivery (DO_2_)/oxygen consumption (VO_2_) uncoupling, eventually leading to metabolic acidosis [1].

This syndrome is defined by a cardiac index (CI) lower than 2 L/min/m^2^, in association with signs of tissue hypoperfusion (cold extremities, mottling, oliguria, high lactate levels) but with no signs of hypovolemia, requiring inotropic drugs or mechanical circulatory support, i.e., extracorporeal membrane oxygenation (ECMO) [2,3].

LCOS is most frequently observed in patients undergoing cardiopulmonary bypass (CPB) during cardiac surgery for the correction of a congenital heart disease. The literature reports a 25–60% incidence of LCOS in pediatric cardiac surgery patients [3,4,5].

Causes of LCOS can include myocardial ischemia due to aortic cross-clamping, residual effects of cardioplegia, CPB-induced myocardial dysfunction, and inflammatory pathway activation due to the exposure of blood to foreign surfaces during CPB [6].

Early recognition and treatment of LCOS are crucial in order to minimize perioperative-related morbidity and mortality.

CI measurement can result in difficulty in pediatric patients through thermodilution due to both the device size and the peculiar cardiovascular pathophysiology of little patients with congenital heart disease, hence the interest to define a reliable marker of LCOS in this subgroup of patients. Another aspect that must be considered in this type of patient, which adds difficulties in CI measurement, is that the definition of “pediatric heart defects” encompasses a large spectrum of pathologies with different physiopathological implications. These are normally divided into two big categories considering the clinical presence of cyanosis and the characteristics of pulmonary circulation: cyanotic and acyanotic defects. Table 1 summarizes the principal defects.

A few recent studies [7,8] have identified the veno-arterial carbon dioxide difference/arterial-venous oxygen difference ratio [P(v-a)CO_2_/C(a-v)O_2_] as an index of tissue hypoxia caused by acute circulatory failure. The physiological rationale is that, according to Fick’s equation, oxygen consumption (VO_2_) and CO_2_ production (VCO_2_) are proportional to cardiac output. Under normal conditions, VCO_2_, VO_2_, and venous to arterial CO_2_ content (CvCO_2_-aCO_2_) and arterial to venous O_2_ content (CaO_2_-CvO_2_) are comparable. Hence, VCO_2_ should not exceed O_2_ production, and the respiratory exchange ratio should not exceed 1. The relationship appears as follows: CO × (CvCO_2_ − CaCO_2_)/CO × (CaO_2_ − CvO_2_).

Since cardiac output is present in both the numerator and denominator the ratio is: C(v-a)CO_2_/C(a-v)O_2_. In steady-state conditions, is it possible to replace CO_2_ concentrations with partial pressure obtaining the P(v-a)CO_2_/C(a-v)O_2_ ratio.

It has been speculated that this ratio could be used as a marker of global tissue hypoxia in adult patients undergoing cardiac surgery. The authors demonstrated that this ratio might be used as a prognostic tool with better relevance when compared to other commonly used perfusion indexes (lactates, central venous oxygen saturation [ScVO_2_], Oxygen Extraction Ratio [ERO_2_], P(v-a)CO_2_, anion gap, prolonged capillary refill time). A ratio higher than 1.6 mmHg/mL is considered predictive of oxygen supply dependency [7,8]. 

The aim of this study was to evaluate the possible use of the P(v-a)CO_2_/C(a-v)O_2_ ratio as a marker of tissue hypoxia in pediatric cardiac patients undergoing cardiac surgery and/or requiring ECMO.

## 2. Materials and Methods

This study included pediatric patients (0–14 years of age) who had undergone cardiac surgery with or without postcardiotomy mechanical support between June 2018 and October 2020 and who were subsequently admitted to the Cardiothoracic Surgery Intensive Care Unit at the University Hospital Integrated Trust of Verona, Italy. All subjects belonged to the REINSURE-ARDS registry, a prospective registry of patients requiring intensive care unit (ICU) admission for any form of respiratory failure (Prog 1946CESC, Prot 72485 12 November 2018, amendment 26 February 2021). Patient identification remained anonymous, and all participants’ legal representatives provided informed consent before inclusion in the registry and for the use of their clinical and biological data.

No exclusion criteria were identified.

Cardiac surgery procedures include either full correction operations and temporary or long-term palliative solutions.

The following data were collected:

- Age and anthropometric measurements (weight and length);
- Type of congenital heart disease (both cyanotic and acyanotic) and type of surgery;
- Pre- and post-surgery laboratory tests (serum creatinine and hemoglobin);
- Intraoperative data (length of CPB, need for transfusions, renal and cerebral near-infrared spectroscopy [NIRS], and body temperature);
- Post-operative data (hemodynamic instability, renal and cerebral near-infrared spectroscopy [NIRS] and body temperature, vasopressor/inotrope infusion, need for mechanical circulation support).

Blood gas analysis both from the arterial catheter and the central venous line were collected:

- At anesthesia induction (pre-CPB).
- At CPB discontinuation.
- Upon ICU admission.
- Before extubation.
- After extubation.
- Before ICU discharge.
- At any significant hemodynamic change (patient needing fluid and/or pharmacologic therapy).

At the time of blood gas collection, body temperature and both renal and cerebral NIRS values were also recorded. 

Arterial and central venous blood gas samples were analyzed using a point-of-care gas analyzer (GEM 4000, Instrumentation Laboratory, Bedford, MA, USA)

Five markers of tissue perfusion were taken into account: lactate, ScVO_2_, ERO_2_, ΔP(v-a)CO_2,_ and P(v-a)CO_2_/C(a-v)O_2_ ratio.

The value of [P(v-a)CO_2_/C(a-v)O_2_] is calculated from the O_2_-derived and CO_2_-derived variables. The calculation formulas are as follows:

CaO_2_ = SaO_2_ × Hb × 1.34 + PaO_2_ × 0.0031

CVO_2_ = ScVO_2_ × Hb × 1.34 + PVO_2_ × 0.0031

C(a-v)O_2_ = CaO_2_ − CvO_2_

P(v-a)CO_2_ = PvCO_2_ − PaCO_2_

P(v-a)CO_2_/C(a-v)O_2_ = (PcvCO_2_ − PaCO_2_)/(CaO_2_ − CcVO_2_)

The primary outcome was to assess the ability of the P(v-a)CO_2_/C(a-v)O_2_ ratio to predict tissue hypoxia in the pediatric cardiac population. The secondary outcome was to evaluate the relationship of other 5 different perfusion markers at different sampling times with the development of LCOS.

The incidence of LCOS was evaluated and defined by hemodynamic instability with the need for high doses of vasopressors/inotropes (defined as milrinone > 0.25 mcg/kg/min, dopamine > 3 mcg/kg/min, adrenaline > 0.05 mcg/kg/min, dobutamine > 5 mcg/kg/min, noradrenaline > 0.1 mcg/kg/min).

Statistically significant differences were evaluated with Fisher’s exact test for nominal variables.

A Wilcoxon signed-rank test (Mann–Whitney) was also used to evaluate the relationship between markers and LCOS development. A non-parametric test was chosen due to the asymmetrical distribution of variables. Main effects and interaction plot graphs were used to complete the analysis. The statistical analysis was performed with Minitab 19.

## 3. Results

The study included 98 pediatric patients undergoing corrective cardiac surgery for congenital heart defects. 

Table 2 shows the demographic data of the population. 

The mean age, expressed in months, was 32.0 ± 48.0 SD (standard deviation). 

All congenital heart defects (CHD) were included in the study. In our sample, 35 patients were affected by a cyanotic heart defect and 63 patients by an acyanotic heart defect (Table 3).

LCOS was detected in 46.9% cases. The incidence of LCOS was significantly higher in cyanotic heart defects compared to non-cyanotic ones (74.2% vs. 31.7%, *p* value < 0.05).

The concentrations of five different perfusion markers at the different sampling times were evaluated as well as their relationship with the development of LCOS, both in the whole studied population and in the two sub-groups (cyanotic and acyanotic CHD).

### 3.1. P(v-a)CO_2_/C(a-v)O_2_ Ratio

**Figure 1:**
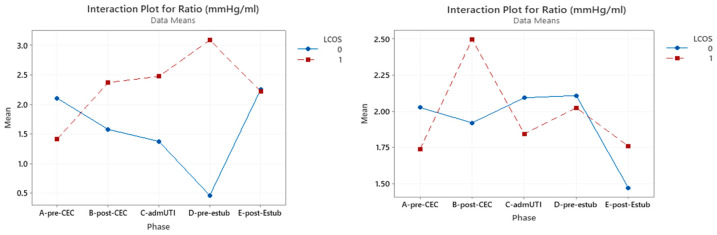
Interaction plot for P(v-a)CO_2_/C(a-v)O_2_ in relation to Low Cardiac Output Syndrome (LCOS) onset (0 = no development of LCOS; 1 = development of LCOS) in the two subgroups of cyanotic (**left**) and acyanotic (**right**) at different sampling times. Abbreviations: A-pre-CEC: at anesthesia induction (pre-CPB), B-post-CEC: at CPB discontinuation, C-admUTI: upon intensive care unit admission, D-pre-estub: before extubation, E-post-Estub: after extubation.

P(v-a)CO_2_/C(a-v)O_2_ ratios only have a statistically significant effect in the whole group in the pre-CBP phase (*p* = 0.023) (Figure 1).

Nonetheless, an interaction was found between the onset of LCOS and the pre-CPB and post-CPB phases, with a 31% increase in patients who developed LCOS and a 17% decrease in patients who do not develop LCOS (Table 4). 

### 3.2. Serum Lactates

Table S1 (Supplementary Materials) illustrates the correlation between serum lactate levels and LCOS development in the whole population, which was significant pre-CPB (*p* = 0.048), post-CBP (*p* = 0.001), and at ICU admission (*p* = 0.026). In the cyanotic CHD group, a statistical significance was reached only pre-CPB (*p* = 0.001), while this was found only post-CPB (*p* = 0.013) in the acyanotic CHD group.

Serum lactates tend to be higher in patients with LCOS and for the cyanotic CHD group. This difference is statistically significant until ICU admission only in the entire population group.

**Figure 2:**
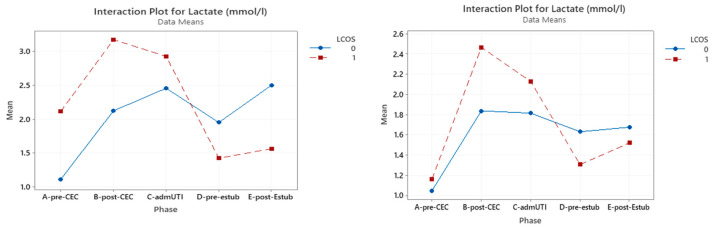
Interaction plot for lactate in relation to Low Cardiac Output Syndrome (LCOS) onset (0 = no development of LCOS; 1 = development of LCOS) in the two subgroups cyanotic (**left**) and acyanotic (**right**) at the different sampling times. Abbreviations: A-pre-CEC: at anesthesia induction (pre-CPB), B-post-CEC: at CPB discontinuation, C-admUTI: upon intensive care unit admission, D-pre-estub: before extubation; E-post-Estub: after extubation.

Figure 2 shows the interaction plot of lactate level in relation to LCOS onset, subpopulation (cyanotic CHD and acyanotic CHD), and sampling time.

### 3.3. ScVO_2_

A significant correlation was found between ScVO_2_ and LCOS development pre-CPB (*p* < 0.0001) and after extubation (*p* = 0.05).

**Figure 3:**
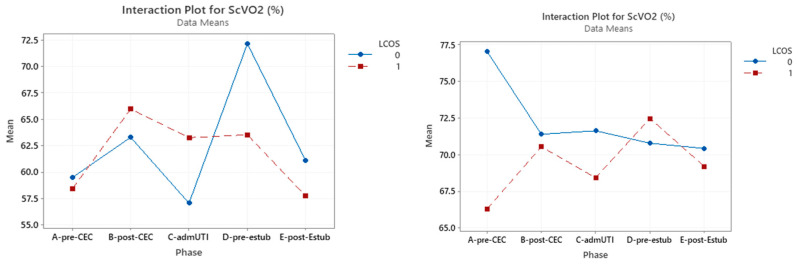
Interaction plot for ScVO_2_ in relation to Low Cardiac Output Syndrome (LCOS) onset (0 = no development of LCOS; 1 = development of LCOS) in the two subgroups cyanotic (**left**) and acyanotic (**right**) at the different sampling times. Abbreviations: A-pre-CEC: at anesthesia induction (pre-CPB), B-post-CEC: at CPB discontinuation, C-admUTI: upon intensive care unit admission, D-pre-estub: before extubation; E-post-Estub: after extubation.

In the cyanotic group, this marker did not reach statistical significance at any evaluated time, while this was reached in the acyanotic group in the pre-CPB sampling *(p* = 0.002) (Table 5) (Figure 3).

### 3.4. ERO_2_

**Figure 4:**
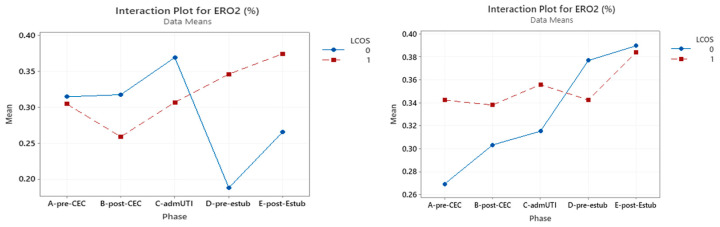
Interaction plot for ERO_2_ in relation to Low Cardiac Output Syndrome (LCOS) onset (0 = no development of LCOS; 1 = development of LCOS) in the two subgroups cyanotic (**left**) and acyanotic (**right**) at the different sampling times. Abbreviations: A-pre-CEC: at anesthesia induction (pre-CPB), B-post-CEC: at CPB discontinuation, C-admUTI: upon intensive care unit admission, D-pre-estub: before extubation; E-post-Estub: after extubation.

Within the whole studied population, only pre-CPB reached statistical significance for prediction of LCOS development (*p* = 0.027). This was confirmed in LCOS patients of the acyanotic group (*p* = 0.013), but ERO_2_ did not have a significant impact in the cyanotic CHD group (Table 6) (Figure 4).

### 3.5. ΔP(v-a)CO_2_

**Figure 5:**
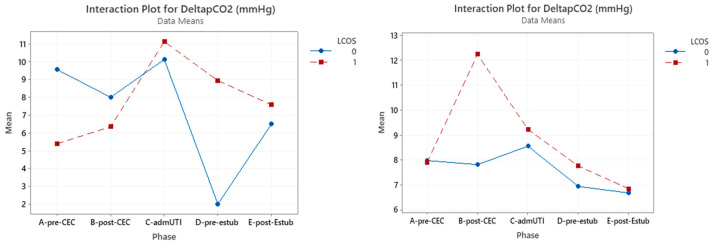
Interaction plot for ∆P(v-a)CO_2_ in relation to Low Cardiac Output Syndrome (LCOS) onset (0 = no development of LCOS; 1 = development of LCOS) in the two subgroups cyanotic (**left**) and acyanotic (**right**) at the different sampling times. Abbreviations: A-pre-CEC: at anesthesia induction (pre-CPB), B-post-CEC: at CPB discontinuation, C-admUTI: upon intensive care unit admission, D-pre-estub: before extubation; E-post-Estub: after extubation.

ΔP(v-a)CO_2_ was significant in pre-CBP considering the whole group (*p* = 0.034).In the cyanotic group only in the pre-CBP phase a statistically significance was reached (*p* = 0.018), while in the acyanotic group this result was obtained in the post-CBP phase (*p* = 0.013) (Table 7) (Figure 5).

## 4. Discussion

Organ perfusion monitoring is crucial in the management of acute circulatory failure and its evaluation needs to be both clinical and biological. The ideal marker of organ perfusion should be quick, not invasive, and easy to measure. The aim of this study was to assess the possible role of some commonly used perfusion markers (such as serum lactate and ScVO_2_) and some others more seldom used (ERO_2_, ΔP(v-a)CO_2_, ratio ΔP(v-a)CO_2_/ΔC(a-v)O_2_) to identify the onset of LCOS and of an anaerobic state. 

Early recognition and treatment of LCOS are crucial in order to avoid ischemic tissue damage and multiorgan failure. 

Serum lactate concentrations are frequently used to detect an anaerobic state, but their rise is often delayed and therefore inadequate. Lactate levels are often inappropriate in the cardiac surgery setting as hyperlactatemia may occur as a consequence of surgical stress, use of beta-adrenergic drugs [9], or lactate lung production [10]. Within surgical stress, lactate washout is indeed reduced and production is enhanced by the anaerobic glycolysis pathway [11]. Moreover, the surgery-related blood flow improvement, especially for renal and coronary perfusion, is associated with higher lactate washout with consequent increased serum levels [12,13]. Nonetheless, high and persistent lactate levels are not justified by these mechanisms. 

This study shows that increased serum lactate concentrations are associated with the development of LCOS in the pre- and after-CPB and ICU admission samples. Therefore, we can define lactate as a reliable LCOS predictor, even though interaction curves might suggest an influence of CPB.

ScVO_2_ is another commonly used marker of tissue hypoxia. This marker has been widely used in pediatric cardiac surgery for a long time, but its use is limited by residual interatrial or intraventricular shunt. We observed a significant association between LCOS and ScVO_2_ in pre-CPB and post-extubation samples in the whole population and in the pre-CPB in the acyanotic CHD. CPB seems to have a different impact on ScVO_2_ values according to the child’s cardiopathy, decreasing in the acyanotic group and increasing in the cyanotic group. This might be ascribable to an improved perfusion in the cyanotic group. 

ERO_2_ expresses the relationship between DO_2_ and VO. When CO is significantly reduced (e.g., blood loss, tamponade, anemia, or hypoxia), an increase in ERO_2_ allows VO_2_ to remain stable until DO_2_ lowers to the critical cut-off and an anaerobic state is initiated. We found indeed that LCOS was significantly related to this marker only pre-CPB within the whole population and in the acyanotic cohort. 

Recently, Δ(v-a) CO_2_ has been proposed as a marker of hypoperfusion [14]. It correlates with CO in adult critically ill patients, with its increase associated with lower CO and higher morbidity [15]. Little is known about the utility of ΔPCO_2_ in the monitoring of pediatric patients after cardiac surgery. In pediatric patients, a relationship between ΔPCO_2_ and ScVO_2_ has been demonstrated after the complete reparation of cardiac defects [16]. Rhodes et al. recently studied the ability of ΔPCO_2_ to predict a poor outcome associated with LCOS in children, concluding that this marker correlates with both cardiac output and oxygen delivery, and it may identify patients at risk of important morbidity immediately upon admission to the ICU [17]. Within our study, ΔPCO_2_ could predict LCOS in the whole population and in the cyanotic CHD group in the pre-CBP phase. It was also significantly associated with LCOS in the post-CPB sample in the acyanotic group. Therefore, it might be an important prognostic tool in this specific group of patients.

ΔP(v-a)CO_2_/ΔC(a-v)O_2_ has been demonstrated to be more sensitive than serum lactate levels thanks to the shorter timeframe for signs of hypoperfusion, and more specifically, as it can detect situations leading to an increase in serum lactates not depending on hypoxia. [15] Nevertheless, it was not a reliable marker of anaerobic metabolism in our pediatric cardiac population and, therefore, this ratio could not be suggested to predict LCOS in this setting. Our findings are not aligned with the current available literature: Monnet et al. [7] and Du et al. [8] demonstrated that this ratio is a marker of anaerobic metabolism, hence apt to predict an adequate response to a DO_2_ challenge. However, these studies did not evaluate the ratio as a prognostication marker for LCOS onset. 

Patel et al. [18] considered ΔP(v-a)CO_2_/ΔC(a-v)O_2_ as a tool to predict the onset of anaerobic metabolism during CPB. Similarly, Mekontso et al. [19] showed its ability to detect anaerobic metabolism in a more reliable way when compared to other indexes (SaO_2_, ScVO_2_, ERO_2_, ΔPCO_2_), but none of them investigate its ability to predict and/or detect LCOS. Recently, Dubin et al. [20] found that ΔP(v-a)CO_2_/ΔC(a-v)O_2_ is not a reliable marker of anaerobic metabolism in the case of hemodilution. Due to their reduced circulating blood volume and to the need to pre-fill the bypass circuit, pediatric patients are potentially more exposed than adults to hemodilution.

Our results should be interpreted in light of this last finding. Indeed, the ratio’s inability to adequately reflect a reduction in blood flow might be due to the fact that in a situation of hemodilution, this marker is primarily influenced by hemoglobin rather than by anaerobiosis. 

Despite not being significant, patients with low CO during CPB presented higher ratio values and, in these patients, an increasing trend can also be observed in the following phases, suggesting hypoperfusion during and after CBP. This means that the increase in ratio values during and following CPB can suggest that the patient might be poorly perfused. 

Serum lactates are also confirmed to be a good marker of hypoperfusion at the end of CPB and at ICU admission, even though its trend might be amplified by CPB itself.

On the other hand, ScVO_2_ is a reliable indicator of low perfusion even after extubation. 

The complexity of hemodynamic monitoring in patients following cardiac surgery and its need to embrace multiple sets of parameters rather than just following a single biomarker trend has recently been studied by Hong et al. [21] who successfully developed several machine learning models to predict LCOS onset after surgery. In his study, he demonstrated that as many as 11 parameters (between biomarkers, echocardiography, and clinical indicators) were necessary to successfully predict LCOS onset in a cohort of adult patients undergoing cardiac surgery. 

Another recent study [22] demonstrated the positive correlation between a score composed of eight items (namely, ejection fraction, oliguria, fluid bolus need, capillary refill time, inotrope requirements, arterial lactates, and cerebral NIRS) and the length of hospital stay, ICU length of stay, vasoactive-inotropic score, lactate mean, and aortic clamp duration in a population of 54 pediatric patients following cardiac surgery. 

One single marker cannot detect hemodynamic instability and the evaluation of a critical patient must include a comprehensive approach (clinical course, vital signs, hemodynamic and tissue perfusion monitoring). For instance, after cardiac surgery, hyperlactatemia is not exclusively a sign of only anaerobic metabolism, and neither do adequate values of ScVO_2_ show that resuscitation is being conducted adequately with absolute certainty. The ratio could be a useful parameter for physicians to detect LCOS only if considered together with clinical, laboratory, and instrumental data collected during hemodynamic alterations in a critical care setting. 

Moreover, the choice of anticoagulation strategy during CPB or ECMO support could also play a role in defining the severity of hypoperfusion, affecting the development of complications. In the pediatric population, the use of bivalirudin compared to heparin seems to be able to reduce the risk of bleeding and thrombosis [23]. This could guarantee a better hemodynamic stability, with a reduced need for resuscitation therapies and an improved microcirculatory perfusion. Recently, the use of Nafamostat Mesylate (NM), a new regional anticoagulant, has been proposed during ECMO assistance in pediatric and adult patients who had evidence of adverse events due to heparin therapy (resistance, heparin-induced thrombocytopenia, clinically uncontrolled bleeding) [24]. This serine protease has shown pleiotropic effects like the ability, when administered before myocardial reperfusion in animal experimental models of cardiac infarction, to modulate the inflammatory response and reduce myocardial damage. The suppression of both complement-mediated response and neutrophil activation was associated with an advantage in terms of survival in ECMO patients treated with NM. Considering this evidence, it could be interesting to understand if the choice of the anticoagulation strategy could, in fact, influence the onset of LCOS, identified through common markers of tissue perfusion.

This study has some limitations. First, the study was conducted on a small convenient sample of patients (even though comparable to other studies in the field) who presented a large variability in age and underlying cardiac defects.

Second, only patients who underwent open heart surgery with the use of CPB were studied. Third, practice variability with respect to sedative and catecholamine administration may represent a confounder. 

Finally, even if we have taken into account the capacity of five markers for predicting LCOS development, this study has not evaluated the capacity of capillary refill time (CRT) in predicting the onset of organ dysfunction. Capillary refill time is defined as the “time required for return of color after application of blanching pressure to a distal capillary bed”. Nowadays, a lot of the literature evidence [25,26] suggests that a prolonged CRT (>3 s) could be used as a simple, rapid, reproducible, and non-invasive parameter with a better ability than hemodynamic macroparameters in identifying microcirculatory hypoperfusion, predicting the severity of hemodynamic instability and the response to resuscitation therapies both in sepsis and cardiogenic shock. Even if it has been demonstrated as effective as a conventional marker in adults, CRT presents some critical procedural aspects; for instance, the site of measurement, the access to this site in many circumstances (for example surgery), and the position in which we have to perform it. These limitations could be even more exacerbated in the pediatric population. For these reasons and considering that we do not currently have guidelines that standardize its execution, its measurement is not a common clinical practice in the ICU.

## 5. Conclusions

ΔP(v-a)CO_2_/ΔC(a-v)O_2_ is not able to predict LCOS onset in pediatric patients undergoing cardiac surgery, despite a rising trend during CPB in patients who then develop LCOS. In addition, raised serum lactate concentrations, reduced ScVO_2_, and increased ERO_2_—measured in the pre-CPB phase—were significantly associated with LCOS onset, thus suggesting them as reliable low perfusion indexes before CPB. 

We can conclude that in the evaluation of a cardiac pediatric patient, a global approach is always to be preferred, taking into consideration clinical data and invasive and non-invasive monitoring. A comprehensive vision allows for a better outcome prediction compared to single values, despite their accuracy.

## Figures and Tables

**Table 1:** Principal cardiac malformations. Abbreviations: AS: aortic stenosis; ASD: atrial septum defect; AVSD: atrioventricular septum defect; COA: coarctation; d-TGV: transposition of great arteries; PDA patent ductus arteriosus; TAPVR: total anomalous pulmonary venous return; TOF: tetralogy of fallout.

Pulmonary Flow	Acyanotic	Cyanotic
Augmented	Left to right shunt: VSD, PDA, ASD, AVSD	Commistion lesions: d-TGA, TAPVR
Normal	Obstructive lesions AS, PS, COA, Cardiomyopathies	-
Reduced	-	Pulmonary flow obstruction with septal defect: TOF, tricuspidal atresia, Ebstein malformation

**Table 2:** Patients’ Demographic Data.

Sex	Male	56.1% (*n* = 55)	
	Female	43.9% (*n* = 43)	
Age (med ± SD)	32.0 ± 48.0 months	Male	35.9 ± 51.1 months
		Female	27.0 ± 34.7 months
Height (med ± SD)	82.9 ± 35.4 cm	Male	85.0 ± 37.7 cm
		Female	78.0 ± 32.1 cm
Weight (med ± SD)	12.7 ± 13.8 kg	Male	13.9 ± 14.9 kg
		Female	11.0 ± 12.1 kg

**Table 3:** Incidence of the different congenital heart defects and related incidence of Low Cardiac Output Syndrome (LCOS).

Population (*n* = 98)	LCOS
Acyanotic, *n* (%): 63 (62%)	20 (31.7%)
Cyanotic, *n* (%): 35 (38%)	26 (74.2%)

**Table 4:** P(v-a)CO_2_/C(a-v)O_2_ values (mmHg/mL) at the different sampling times correlated with the presence/absence of Low Cardiac Output Syndrome (LCOS). Abbreviations: LCOS: Low Cardiac Output Syndrome, CPB: Cardiopulmonary bypass, ICU: intensive care unit, SD: standard deviation, CHD: congenital heart defect.

		Whole Population		Cyanotic CHD		Acyanotic CHD	
Sampling Times	LCOS	Mean Value (SD) mmHg/mL	*p*-Value	Mean Value (SD) mmHg/mL	*p*-Value	Mean Value (SD) mmHg/mL	*p*-Value
Pre-CPB	Yes	1.55 (1.00)	0.023	1.41 (0.85)	0.131	1.74 (1.18)	0.286
	No	2.05 (1.05)		2.10 (1.22)		2.06 (1.02)	
Post-CPB	Yes	2.44 (2.94)	0.5	2.40 (3.79)	0.377	2.49 (1.33)	0.167
	No	1.87 (1.26)		1.58 (0.91)		1.92 (1.30)	
ICU-adm.	Yes	2.21 (1.67)	0.256	2.47 (2.06)	0.160	1.84 (0.82)	0.990
	No	1.95 (1.64)		1.37 (0.94)		2.09 (1.75)	
Pre-ext.	Yes	2.61 (4.41)	0.770	3.09 (5.86)	-	2.02 (1.24)	0.958
	No	2.04 (1.53)		0.45 (-)		2.11 (1.53)	
Post-ext.	Yes	2.04 (1.74)	0.239	2.22 (1.99)	0.99	1.76 (1.32)	0.586
	No	1.52 (1.41)		2.25 (2.22)		1.47 (1.39)	

**Table 5:** ScVO_2_ values (mmol/L) at the different sampling times correlated with the presence/absence of Low Cardiac Output Syndrome (LCOS). Abbreviations: LCOS: Low Cardiac Output Syndrome, CPB: cardiopulmonary bypass, ICU: intensive care unit, SD: standard deviation, CHD: congenital heart defect.

		Whole Population		Cyanotic CHD		Acyanotic CHD	
Sampling Times	LCOS	Mean Value (SD) mmol/L	*p*-Value	Mean Value (SD) mmol/L	*p*-Value	Mean Value (SD) mmol/L	*p*-Value
Pre-CPB	Yes	61.83 (14.73)	0.000061	58.41 (14.63)	0.792	66.28 (13.98)	0.002
	No	74.06 (14.73)		59.48 (13.99)		77.03(9.82)	
Post-CPB	Yes	67.89 (12.44)	0.197	65.86 (12.95)	0.919	70.53 (11.55)	0.652
	No	70.59 (14.39)		63.29 (22.26)		71.38 (12.57)	
ICU-adm.	Yes	65.36 (15.78)	0.224	63.25 (17.63)	0.345	68.41 (12.52)	0.309
	No	69.01 (13.79)		57.04 (15.49)		71.62 (11.82)	
Pre-ext.	Yes	67.04 (15.36)	0.301	63.52 (17.62)	0.689	72.45 (9.25)	0.940
	No	70.86 (14.00)		72.20 (23.50)		70.77 (13.77)	
Post-ext.	Yes	62.17 (15.85)	0.05	57.74 (16.78)	0.848	69.18 (11.73)	0.654
	No	69.61 (12.54)		61.07 (16.67)		70.41 (12.12)	

**Table 6:** ERO_2_ values (%) at the different sampling times correlated with the presence/absence of Low Cardiac Output Syndrome (LCOS). Abbreviations: LCOS (Low cardiac output syndrome), CPB (Cardiopulmonary bypass), ICU (Intensive Care Unit), SD (standard deviation), CHD (congenital heart defect).

		Whole Population		Cyanotic CHD		Acyanotic CHD	
Sampling Times	LCOS	Mean Value (SD)%	*p*-Value	Mean Value (SD)%	*p*-Value	Mean Value (SD)%	*p*-Value
Pre-CPB	Yes	32.1 (14.4)	0.027	30.4 (15.4)	0.938	43.2 (12.5)	0.013
	No	27.6 (12.1)		31.4 (13.1)		26.9 (11.8)	
Post-CPB	Yes	29.3 (15.1)	0.874	25.6 (13.2)	0.542	33.8 (16.4)	0.403
	No	30.1 (14.7)		31.7 (21.2)		30.3 (13.6)	
ICU-adm.	Yes	32.6 (17.2)	0.580	30.6 (17.4)	0.375	35.6 (17.0)	0.223
	No	32.3 (18.4)		36.9 (15.9)		31.5 (18.8)	
Pre-ext.	Yes	34.4 (22.7)	0.921	34.6 (23.5)	0.309	34.2 (22.5)	0.769
	No	34.2 (22.2)		18.8 (16.8)		37.7 (25.1)	
Post-ext.	Yes	37.8 (23.6)	0.549	37.4 (23.3)	0.340	38.4 (24.9)	0.902
	No	36.2 (23.0)		26.6 (9.1)		38.9 (25.7)	

**Table 7:** Delta P(v-a)CO_2_ values (mmHg) at the different sampling times correlated with the presence/absence of Low Cardiac Output Syndrome (LCOS). Abbreviations: LCOS (Low cardiac output syndrome), CPB (Cardiopulmonary bypass), ICU (Intensive Care Unit), SD (standard deviation), CHD (congenital heart defect).

		Whole Population		Cyanotic CHD		Acyanotic CHD	
Sampling Times	LCOS	Mean Value (SD) mmHg	*p*-Value	Mean Value (SD) mmHg	*p*-Value	Mean Value (SD) mmHg	*p*-Value
Pre-CPB	Yes	6.49(4.71)	0.034	5.39 (3.67)	0.018	7.89 (5.57)	0.734
	No	8.27 (3.92)		9.56 (4.50)		7.97 (3.71)	
Post-CPB	Yes	8.25 (5.11)	0.837	6.24 (3.94)	0.512	12.24 (6.93)	0.013
	No	8.26 (5.21)		8.00 (5.22)		7.80 (3.83)	
ICU-adm.	Yes	10.29 (5.85)	0.303	11.13 (6.46)	0.650	9.22 (4.95)	0.990
	No	8.92 (5.66)		10.13 (7.81)		8.55 (4.99)	
Pre-ext.	Yes	8.43 (6.98)	0.512	8.94 (8.02)	-	7.75 (5.58)	0.646
	No	7.00 (5.05)		2.00 (-)		6.93 (5.13)	
Post-ext.	Yes	6.85 (5.86)	0.734	7.58 (6.79)	0.99	7.89 (5.57)	0.734
	No	6.96 (5.71)		6.50 (4.95)		7.97 (3.71)	

## Data Availability

The data presented in this study are available from the corresponding author on request.

## Supplementary Materials

The following are available online at https://www.mdpi.com/article/10.3390/jcm12175700/s1: Table S1: illustrates the correlation between serum lactate levels and LCOS in the whole population.
