# Supplementary material for: Analysis of P(v-a)CO2/C(a-v)O2 Ratio and Other Perfusion Markers in a Population of 98 Pediatric Patients Undergoing Cardiac Surgery
Source: J Clin Med. 2023 Sep 1;12(17):5700. doi: 10.3390/jcm12175700 (PMC10488867; doi:10.3390/jcm12175700)
Supplement: Supplementary file 1 [file jcm-12-05700-s001.zip › jcm-2577830-supplementary.pdf]

Table S1. Correlation between serum lactate levels and LCOS development in the whole population.

Sampling Times	LCOS	Mean Value (sd) mmol/L	*p*-Value
**Pre-CPB**	Yes	1.71 (2.89)	0.048
	No	1.05 (0.35)	
**Post-CPB**	Yes	2.90 (2.62)	0.001
	No	1.87 (0.87)	
**ICU-adm.**	Yes	2.60 (2.59)	0.026
	No	1.91 (1.17)	
**Pre-ext.**	Yes	1.37 (0.57)	0.234
	No	1.68 (0.94)	
**Post-ext.**	Yes	1.54 (0.71)	0.403
	No	1.79 (1.10)	
